# Point-of-Care-ultrasound in undergraduate medical education: a scoping review of assessment methods

**DOI:** 10.1186/s13089-023-00325-6

**Published:** 2023-06-11

**Authors:** Celina DeBiasio, Paul Pageau, Allan Shefrin, Michael Y. Woo, Warren J. Cheung

**Affiliations:** 1 Division of Dermatology, Ottawa Hospital and University of Ottawa, Ottawa, ON Canada; 2 Department of Emergency Medicine, University of Ottawa, Ottawa Hospital, 1053 Carling Avenue Ottawa, Ottawa, ON Canada; 3 Division of Emergency Medicine, Department of Pediatrics, Children’s Hospital of Eastern Ontario, University of Ottawa, Ottawa, ON Canada; 4 Ottawa Hospital Research Institute, Ottawa, ON Canada

**Keywords:** POCUS, Undergraduate Medical Education, Assessment

## Abstract

**Background:**

Point-of-Care-Ultrasound (POCUS) curricula have rapidly expanded in undergraduate medical education (UME). However, the assessments used in UME remain variable without national standards. This scoping review characterizes and categorizes current assessment methods using Miller’s pyramid for skills, performance, and competence of POCUS in UME.

A structured protocol was developed using the Preferred Reporting Items for Systematic Reviews and Meta-Analyses Extension for Scoping Reviews (PRISMA-ScR). A literature search of MEDLINE was performed from January 1, 2010, to June 15, 2021. Two independent reviewers screened all titles and abstracts for articles that met inclusion criteria. The authors included all POCUS UME publications in which POCUS-related knowledge, skills, or competence were taught and objectively assessed. Articles were excluded if there were no assessment methods used, if they exclusively used self-assessment of learned skills, were duplicate articles, or were summaries of other literature. Full text analysis and data extraction of included articles were performed by two independent reviewers. A consensus-based approach was used to categorize data and a thematic analysis was performed.

**Results:**

A total of 643 articles were retrieved and 157 articles met inclusion criteria for full review. Most articles (*n* = 132; 84%) used technical skill assessments including objective structured clinical examinations (*n* = 27; 17%), and/or other technical skill-based formats including image acquisition (*n* = 107; 68%). Retention was assessed in *n* = 98 (62%) studies. One or more levels of Miller’s pyramid were included in 72 (46%) articles. A total of four articles (2.5%) assessed for students’ integration of the skill into medical decision making and daily practice.

**Conclusions:**

Our findings demonstrate a lack of clinical assessment in UME POCUS that focus on integration of skills in daily clinical practice of medical students corresponding to the highest level of Miller’s Pyramid. There exists opportunities to develop and integrate assessment that evaluate higher level competencies of POCUS skills of medical students. A mixture of assessment methods that correspond to multiple levels of Miller’s pyramid should be used to best assess POCUS competence in UME.

**Supplementary Information:**

The online version contains supplementary material available at 10.1186/s13089-023-00325-6.

## Background

Over the last decade, the integration of Point-of-Care-Ultrasound (POCUS) for clinical screening, diagnosis, and management has rapidly expanded across multiple medical disciplines [1–3]. As a clinical tool, POCUS is easily accessible, portable, and cost-effective [4]. Subsequently, POCUS has also expanded in both post-graduate medical education (PGME) and undergraduate medical education (UME) [3, 5].

Within UME, assessment of POCUS-related skills drives learning and is multipurposed; it serves as a measurement of knowledge acquisition, stimulus for feedback and performance improvement, and as a means of measuring learners’ skill development [6]. While methods of assessment, including multiple-choice questions (MCQs) and technical skill evaluation such as objective structural clinical evaluations (OSCEs) have traditionally been used, an emerging approach of targeting multiple assessment methods to better measure POCUS skills and thereby competency has been suggested in UME [7] and clinical ultrasound in general [8].

In addition to targeting multiple assessments, determining POCUS competence would benefit from an overall programmatic assessment approach [9]. This approach includes collecting ‘routine information about the learner’s competence and progress is continually collected, analyzed and, where needed, complemented with purposively collected additional assessment information, with the intent to maximally inform the learner and their mentor’. [9].

Given the variability in assessment methods used across POCUS UME curricula [3], well-established frameworks such as Miller’s pyramid for clinical assessment may be used for categorization [10]. Miller’s framework is a useful tool for medical educators to aid in correlating learning outcomes with different expectations of a learner’s abilities at various learning stages [10]. Miller’s pyramid is divided into four levels, with the base of the pyramid, ‘knows’, defined by a medical professional’s knowledge of a learned skill, including knowledge-based MCQs [10]. Level 2, ‘knows how’ corresponds to application of knowledge such as problem-solving MCQs, whereas level 3, ‘shows how’ relates to demonstration of a learned skill, including OSCEs. At the top of the pyramid is level 4, which represents a learner’s performance in clinical practice [10, 11]. The highest level of Miller’s pyramid aligns well with the higher O-SCORE entrustability scale measurements [12]. For example, successfully demonstrating performance in the workplace (Miller level 4) corresponds well to the O-SCORE entrustability level 4, ‘I needed to be in the room just in case’ and level 5, ‘I did not need to be there’. Since POCUS is a clinically integrated and largely user-dependent skill [13], the assessment of skills within POCUS UME is critical to a curricula’s success. However, there is little published regarding what assessments are currently used within UME, as well as an absence of nationally adopted standards or guidelines for POCUS assessment.

We performed a scoping review providing a detailed synthesis of the assessment methods implemented in international POCUS UME literature and categorized each assessment into Miller’s framework.

## Methods

### Protocol

Our protocol was based on the Preferred Reporting Items for Systematic Reviews and Meta-analysis Protocols extension for Scoping reviews (PRISMA-ScR) [14]. No patient data were included and the scoping review did not require research ethics board approval.

### Information sources

A librarian assisted search of MEDLINE was conducted from January 1, 2010, to June 15, 2021. We included all articles published since 2010 when ultrasound became more prevalent in medical school curricula [4, 5, 15, 16]. The final MEDLINE search strategy can be found in Additional file 1 Appendix S1.

### Eligibility criteria

**Fig. 1:**
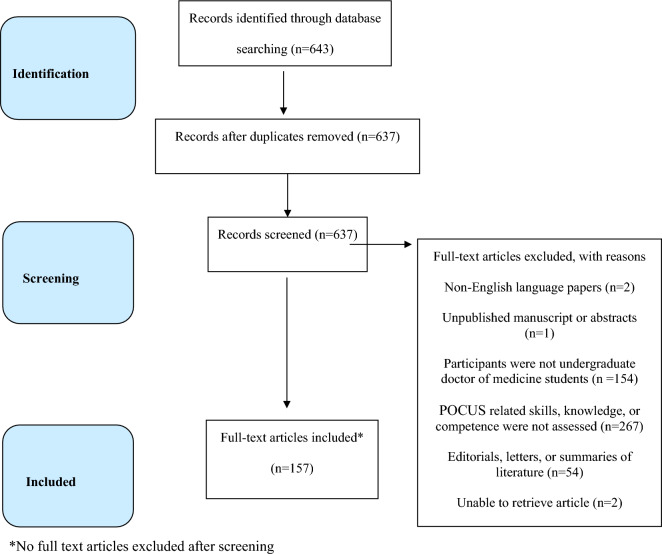
PRISMA-ScR Flow [11]

Inclusion criteria included all English language POCUS UME publications in which POCUS-related knowledge, skills, or competence were taught and objectively assessed. Participants were restricted to both pre-clinical (pre-clerkship) and clinical (clerkship) medical students. Articles were excluded if there were no assessment methods used. Articles that exclusively used self-assessment of learned skills were also excluded. Editorials, letters, scoping reviews, systematic reviews, meta-analyses, or summaries of other literature were excluded. Any duplicate articles were removed. The article exclusion process is depicted in the PRISMA flow diagram (Fig. 1) [14].

### Selection of sources of evidence

Two independent reviewers screened abstracts for inclusion (CD and PP). Any disagreements between the reviewers were resolved by another member of the research team (MW). Articles that met inclusion criteria for full text review were reviewed by the same two independent reviewers (CD and PP). Data were extracted into a standardized data charting form.

### Data charting process

**Fig. 2:**
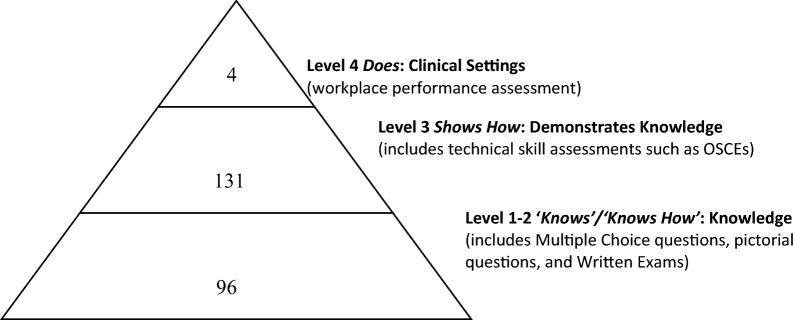
Modified Miller’s Pyramid: Number of assessments in included articles corresponding to Miller’s framework [9]

The process of chart and category development was iterative with multiple revisions to arrive at common themes and categories. Since most of the included articles did not list the MCQs or written questions used or provide sufficient details on the content, level 1 and level 2 of Miller’s framework were combined (Fig. 2) [10]. A standardized data charting form was developed, trialed, and revised prior to data abstraction and calculating Kappa coefficient of agreement. Two reviewers (CD and PP) independently charted the data, discussed results, and attempted to reach consensus. If disagreements occurred during the data charting process, adjudication was made by another member of the research team (MW).

### Data items

Data items such as author, year of publication, study participants, assessment characteristics, assessment methods, and the modified Miller’s pyramid level were abstracted and charted. Level one and two of Miller’s pyramid included any assessment of knowledge through MCQs, short answers, pictorial, or case-based questions [10]. Level three encompassed any assessment that required students to demonstrate a skill they had learned in an artificial setting [10]. This included any technical skill assessments such as image reproduction, scanning a standardized patient or peer, or OSCEs. Level four was defined to include workplace-based assessment methods that assessed students in an authentic clinical environment as a part of the learner’s day-to-day work [10].

## Results

### Selection of sources of evidence

The search yielded 643 titles from 26 countries. The initial agreement between the two independent reviewers for screened abstracts was strong with Cohen’s $\kappa =$ 0.95. After removing duplicates and applying inclusion and exclusion criteria 157 articles met inclusion for a full text review Additional file 2: Appendix S2. Articles predominantly came from the United States (*n* = 64; 41%) and Canada (*n* = 12; 21%). A detailed overview of the selection process is shown in Fig. 1.

### Synthesis of results

#### Medical student learners

The sample sizes of articles ranged from three to 1084. For articles that reported if participants were in their preclinical and/or clinical training (*n* = 130; 83%), 61 (47%) articles included assessments of preclerkship students and 83 (63%) included assessments of clerkship or final year students (Table 1).

**Table 1:** Learners’ Level of training if mentioned compared to Miller’s Pyramid of Assessment

Learners level of training (if mentioned)	Miller pyramid level 1/2^a^ = ‘knows/knows how’ 3 = ‘shows how’ 4 = ‘Does’					
	1/2	3	4	1/2 and 3	3 and 4	Grand Total
Preclinical learners	11	25	1	24	0	61
Clinical learners	10	26	44	1	1	83

Some articles included both preclinical and clinical learners.  N = 27 articles did not specify if participants were in their preclinical or clinical training

^a^Most included articles did not list the MCQs or written questions used or provide sufficient details on the content therefore level 1 and level 2 of Miller’s framework were combined

### Assessment characteristics

The average number of unique assessments used per article was 1.5. Most of the included articles assessed for retention (*n* = 98; 62%). Technical skill examinations such as OSCEs (*n* = 27; 17%) and/or other technical skill-based formats including image acquisition (*n* = 107; 68%) were incorporated in 132 (84%) articles. Approximately 51% (*n* = 80) of articles included knowledge-based assessments such as MCQs, short answers, pictorial, and/or case-based questions. Details of assessment characteristic are described in Table 2.

**Table 2:** Summary of assessment method of published POCUS UME Curricula

Assessment method	Articles with assessment, n
Multiple Choice questions, short answers, pictorial, or case-based questions (assessment of knowledge)	80
OSCE	25
Technical Skill Examination (including producing images) excluding OSCE	105
OSCE and Other Technical Skill Examination (including producing images)	2
Skill assessment on simulator, phantom, animal model, virtual model, or cadaver	50
Assessment with standardized patients or peers	66
Assessment of patients	32
Retention assessed (greater than 1 day)	98

Articles often used more than one assessment method

*POCUS* Point-of-Care-Ultrasound, *UME* Undergraduate Medical Education, *OSCE *Objective Structured Clinical Examination

Four articles (2.5%) used an objective structured assessment of ultrasound skills (OSAUS) for skills evaluation and four (2.5%) used the generalized assessment of the Brightness Mode Quality Ultrasound Imaging Examination Technique (B-QUIET) [2, 14]. Notably, 55 (35%) articles combined technical skill assessments with knowledge-based examinations. Two articles (1.3%) used both an OSCE and another form of objective technical skill examination for assessments of medical students.

For those articles that used technical skill evaluations (*n* = 132; 84%), there was a larger number of articles that assessed skills on a standardized patient and/or peer (*n* = 66; 50%), than compared to those that used a simulator, phantom, animal model, or cadaver (*n* = 50; 38%). Articles that assessed medical learners’ skills with real patients in a clinical context were included in 32 (24%) of articles. In articles that included real patients, 28 (88%) articles pre-selected the patients for learners based on specific existing health conditions.

### Assessment framework

The most frequently reported assessment method was categorized in level 3 of Miller’s pyramid (*n* = 131; 83%). In these articles, medical students were evaluated on their learned POCUS skill using technical skill assessments including OSCEs in an artificial setting. Although some articles included real patients, because they were pre-selected and not part of the trainee’s day-to-day clinical work, these articles were categorized into level 3. The next most frequent method of assessment was categorized in the combined levels 1 and 2 of Miller’s pyramid ‘knows’ and ‘knows how’ (*n* = 96; 61%). Most of these articles (*n* = 74; 77%) used MCQs to assess for knowledge of the learned skills. Almost half (47%) of the studies were completed in pre-clerkship students where assessment of Level 4 of Miller’s pyramid may not be practical.

Only 4 (2.5%) articles reported on assessment methods corresponding to level four of Miller’s pyramid, ‘does’ (4/157 articles) [18–21]. Notably, three (75%) of these articles reported on more than one level of Miller’s pyramid [18, 20, 21]. Two articles (50%) assessed for all four levels of Miller’s pyramid [18, 21]. All four articles (100%) assessed for retention of learned skills and three (75%) involved assessment of clerkship students.

One or more levels of Miller’s pyramid were included in 72 (46%) articles. The most frequently used combination was levels one/two, ‘knows/knows how’ with level three, ‘shows’ (*n* = 71 of 157 articles).

## Discussion

Despite the increasing integration of POCUS within UME, there is a relative paucity of UME POCUS assessment tools that target the highest level of Miller’s pyramid reported in the literature. While assessing lower levels of Miller’s pyramid provides the advantage of ease of evaluation through knowledge-based MCQs and short answers, assessing higher levels of Miller’s pyramid enables more effective assessment of a learner’s competence in their day-to-day clinical work. A recent survey of UME directors demonstrated that the incorporation of questions into course examinations was the most common method of POCUS assessment [22].

Clinical assessment of learned skills allows for multiple subcompetencies of POCUS to be assessed including knowledge, identifying sonographic indications, demonstration of sonographic skills, image interpretation, and medical decision-making [8]. In an article by Olszynski et al. the authors successfully assessed the highest level of Miller’s pyramid in a clinical ultrasonography clerkship elective. Assessment methods were longitudinal and included multiple-choice examinations, technical skill examinations, and clinical assessment forms that were completed by clinical rotation supervisors. The goal of these clinical assessment forms was to assess the appropriateness and reliability of students’ skills in daily clinical practice. In an article by Krause et al. the authors assessed level four of Miller’s pyramid through a daily clinical assessment method in which students were required to complete and record a minimum of three clinically indicated extended Focused Assessment with Sonography in Trauma (eFAST) examinations during their surgical clerkship rotation [21]. An emergency staff physician or resident would then review the learner’s POCUS image and interpretation. At the same time, both of these authors also successfully integrated additional levels of Miller’s pyramid using knowledge-based examinations and technical skill assessments [18, 21]. Notably, one article by Andersen et al. provided limited training on handheld ultrasound devices to students then asked learners to acquire and interpret ultrasound images during their clinical rotations [20]. The images and interpretations were subsequently reviewed by staff physicians. This study demonstrated students ability to acquire and interpret their POCUS images in daily clinical practice with significant accuracy. The integration of handheld ultrasound devices and recording of images would allow a feasible assessment method to inform workplace-based assessments. Clinical indication, interpretation and clinical integration of POCUS images would need to be included in the assessment to provide a more robust evaluation of POCUS use in the workplace. The handheld ultrasound devices have the added advantage of increased accessibility and limited associated costs for UME programs [23].

A challenge associated with targeting Miller’s highest level of clinical assessment is the requirement for access to clinical environments. Due to the differences in medical school training and curricula across North America and even internationally, it may be difficult for pre-clinical learners to gain clinical opportunities prior to their formal clinical training. For these reasons, targeting level one, two, and/or three of Miller’s pyramid in the preclinical years, may be advantageous. The most common assessment method reported in the present scoping review for all articles was evaluation of technical performance. This included standardized assessments such as OSCEs, OSAUS, B-QUIET, and non-standardized tools which involved assessment of POCUS image acquisition skills. POCUS is a user-dependent skill and therefore acquisition and assessment of technical competence is an important component of competency. Standardized assessments such as OSCEs are beneficial in that they provide realistic simulations of patient care in a controlled environment. However, disadvantages associated with OSCE-style assessment methods include cost, time, and reliability of assessments across multiple stations [8]. If not successfully standardized, OSCEs are subject to observer bias and inter-rater agreement [6, 24]. Notably, one article in this review focused on transvaginal ultrasound training and used OSAUS as an objective assessment method while also assigning a global rating scale (GRS) using a five-point Likert scale [25]. While OSAUS provides an objective means of assessment, validity evidence has not yet been collected in the undergraduate medical student population [26].

Ultimately, employing a mixture of assessment methods that correspond to multiple levels of Miller’s pyramid may be the best approach to ensure a feasible and more comprehensive assessment of learned skills [10]. Slightly less than half of the articles from this scoping review used a multi-assessment approach integrating more than one level of Miller’s framework. The most common combination of assessment methods was evaluation of knowledge using MCQs and/or written examinations and evaluation of skills with technical demonstration. Because ultrasound clinical competency is multidimensional, educational models that assess for different subcompetencies are needed in UME. One example of such a model is the I-AIM tool, which stands for ‘indication, acquisition, interpretation, and medical decision making’ [27]. I-AIM is a standardized checklist for assessment of physician-performed focused sonographic examinations. Notably, one article in this scoping review introduced students to the I-AIM technique; however, the learned skills were assessed with written pre and post-knowledge tests rather than direct observation [28]. While the I-AIM model incorporates knowledge, technical skill, and medical decision making of ultrasonography, validity evidence for its use in undergraduate medical students is lacking [27]. The Ultrasound Competency Assessment Tool (UCAT) is another model that integrates multiple levels of Miller’s pyramid into POCUS assessment [29]. The UCAT consists of five domains including preparation, image acquisition, image optimization, clinical integration, and entrustment [29]. While not yet evaluated in the UME population, there is early validity evidence in POCUS competence for post-graduate Emergency Medicine trainees [29].

The future of assessing POCUS competence may benefit from a programmatic assessment approach that includes multiple levels of Miller’s pyramid using standardized and non-standardized methods. These methods can be formative assessments for the learner and then collected and analyzed by a faculty or committee to develop a rich diagnostic picture to allow a defensible, high-stakes decision of POCUS competence.

## Limitations

Despite using an inclusive search strategy developed and conducted with an experienced librarian, our scoping review was limited to one electronic database, thereby limiting the breadth of papers reviewed. Additionally, although much of POCUS curricula has been incorporated into UME within the past decade [3], assessment methods reported in articles published prior to 2010 were not included within the scope of this review. Finally, many articles did not provide sufficient details on the assessment methods used (e.g., MCQs, assessment checklists, scoring rubrics for technical skill assessments, etc.). As a result, categories of assessments in level one and two of Miller’s pyramid were combined, which limited detailed categorization. The majority of articles were from North America which may limit generalizability to international UME.

## Conclusions

This scoping review represents a synthesis of the current published literature of POCUS assessment methods in UME. Our findings demonstrate a lack of clinical ultrasound skills assessment in daily clinical practice of medical students corresponding to the highest level of Miller’s pyramid. A programmatic assessment approach with a mixture of assessment methods that correspond to multiple levels of Miller’s pyramid may be the future of assessing POCUS competence in UME.

## Supplementary Information

**Additional file 1:** Supplement 1, Scoping review search strategy.

**Additional file 2:** Supplement 2, Included studies and Miller's level of assesment.

## Data Availability

All data generated or analyzed during this study are included in this published article and its supplementary information files
