# Supplementary material for: Point-of-Care-ultrasound in undergraduate medical education: a scoping review of assessment methods
Source: Ultrasound J. 2023 Jun 11;15:30. doi: 10.1186/s13089-023-00325-6 (PMC10258183; doi:10.1186/s13089-023-00325-6)
Supplement: Supplementary file 1 — Additional file 1: Supplement 1, Scoping review search strategy. [file 13089_2023_325_MOESM1_ESM.docx]

**Appendix 1:** Search Methods MEDLINE (January 1946 to June 15, 2021)

1 exp Echocardiography/

2 Ultrasonography/

3 cardiac echo.tw,kw.

4 (ultrasound or sonogram or sonograph* or ultra sound or ultrasonograph*).tw,kw.

5 (echocardiog* or pocus).tw,kw.

6 or/1-5

7 Education, Medical, Undergraduate/

8 Students, Medical/

9 Clinical Clerkship/

10 (medical student* or clerk* or medical school* or preclerk* or undergrad*).tw,kw.

11 or/7-10

12 6 and 11

13 educational measurement/

14 clinical competence/

15 competen*.tw,kw.

16 "Surveys and Questionnaires"/

17 (survey* or questionnaire*).tw,kw.

18 (measur* or exam* or test or tests or assess* or evaluat* or scale* or osce).tw,kw.

19 or/13-18

20 12 and 19

21 limit 20 to yr="2010 -Current"

22 (202008* or 202009* or 20201* or 2021*).dt.

23 21 and 22
