# Supplementary material for: Point-of-Care-ultrasound in undergraduate medical education: a scoping review of assessment methods
Source: Ultrasound J. 2023 Jun 11;15:30. doi: 10.1186/s13089-023-00325-6 (PMC10258183; doi:10.1186/s13089-023-00325-6)
Supplement: Supplementary file 2 — Additional file 2: Supplement 2, Included studies and Miller's level of assesment. [file 13089_2023_325_MOESM2_ESM.docx]

Appendix 2: Table of included articles

| First Author | Year of publication | Country | Miller Pyramid (Knows/knows how=1/2, Shows how=3, Does/action=4) |
| --- | --- | --- | --- |
| Afonso | 2010 | USA | 1/2 and 3 |
| Ahmed | 2019 | Ireland | 3 |
| Ahn | 2015 | USA | 3 |
| Alfageme | 2016 | Spain | 1/2 |
| Amini | 2016 | USA | 1/2 and 3 |
| Amini | 2016 | USA | 1/2 |
| Amini | 2015 | USA | 1/2 and 3 |
| Andersen | 2014 | Norway | 3 and 4 |
| Ang | 2018 | Australia | 1/2 and 3 |
| Armson | 2021 | UK | 1/2 and 3 |
| Backhaus | 2018 | Germany | 3 |
| Bahner | 2014 | USA | 1/2 and 3 |
| Bahner | 2012 | USA | 3 |
| Barrington | 2016 | Australia | 1/2 and 3 |
| Bell | 2019 | USA | 1/2 and 3 |
| Bem-Sasson | 2019 | Israel | 3 |
| Bentley | 2015 | USA | 1/2 and 3 |
| Blackstock | 2015 | USA | 1/2 and 3 |
| Britz, V | 2020 | Germany | 3 |
| Brown | 2012 | USA | 1/2 |
| Byrne | 2019 | Switzerland | 1/2 and 3 |
| Cawthorn | 2014 | Canada | 1/2 and 3 |
| Celebi | 2019 | Germany | 1/2 and 3 |
| Celebi | 2019 | Germany | 1/2 and 3 |
| Cevik | 2019 | United Arab Emirates | 1/2 |
| Cevik | 2018 | United Arab Emirates | 3 |
| Cheng | 2014 | Taiwan | 1/2 and 3 |
| Chuan | 2021 | Australia | 3 |
| Coiffier | 2020 | Hong Kong, China | 1/2 and 3 |
| Cook | 2020 | USA | 1/2 |
| Cortez | 2014 | USA | 1/2 and 3 |
| Cruvinel | 2019 | Brazil | 1/2 and 3 |
| Cuca | 2013 | Germany | 1/2 |
| Damewood | 2012 | USA | 1/2 and 3 |
| Davis | 2017 | USA | 3 |
| Di Pietro | 2020 | Italy | 1/2 and 3 |
| Di Pietro | 2018 | Italy | 1/2 and 3 |
| Dinh | 2015 | USA | 3 |
| Dreher | 2014 | USA | 1/2 |
| Dyre | 2017 | Denmark | 3 |
| Ebner | 2019 | Germany | 1/2 and 3 |
| Eimer | 2021 | Germany | 1/2 and 3 |
| Eissa | 2015 | USA | 1/2 and 3 |
| Elison | 2020 | USA | 3 |
| Eroglu | 2018 | Turkey | 1/2 and 3 |
| Etienne | 2021 | France | 3 |
| Favot | 2015 | USA | 1/2 |
| Felipe | 2017 | Spain | 3 |
| Fernandes | 2020 | Netherlands | 1/2 and 3 |
| Florescu | 2015 | Romania | 1/2 and 3 |
| Fodor | 2012 | Romania | 3 |
| Fox | 2017 | USA | 3 |
| Friedman | 2018 | USA | 1/2 |
| Fuchs | 2018 | Israel | 3 |
| Garcia de Casasola Sanchez | 2015 | Spain | 3 |
| Garcia de Casasola Sanchez | 2014 | Spain | 3 |
| Garcia-Casasola | 2016 | Spain | 3 |
| Gogalniceanu | 2010 | UK | 3 |
| Goldstein | 2021 | USA | 1/2 and 3 |
| Gradl-Dietsch | 2018 | Germany | 1/2 and 3 |
| Griffiths | 2017 | UK | 3 |
| Hamza | 2019 | Germany | 1/2 and 3 |
| Heiberg | 2015 | Denmark | 3 |
| Heinzow | 2013 | Germany | 3 |
| Hempel | 2020 | Germany | 1/2 and 3 |
| Hempel | 2016 | Germany | 1/2 and 3 |
| Ho | 2015 | Hong Kong, China | 1/2 and 3 |
| Hoppmann | 2015 | USA | 1/2 and 3 |
| Hu | 2021 | Taiwan | 1/2 and 3 |
| Huppe | 2019 | Germany | 3 |
| Jeppesen | 2012 | USA | 1/2 and 3 |
| Khoury | 2021 | Canada | 1/2 and 3 |
| Kim | 2017 | Korea | 1/2 |
| kim | 2014 | Germany | 3 |
| Knobe | 2012 | Germany | 1/2 and 3 |
| Knobe | 2010 | Germany | 1/2 and 3 |
| Knudsen | 2018 | Germany | 1/2 |
| Kobal | 2017 | Israel | 1/2 and 3 |
| Kopac | 2013 | Canada | 3 |
| Krause | 2017 | USA | 1/2, 3, and 4 |
| Kuhl | 2012 | Germany | 3 |
| Kukulski | 2018 | USA | 1/2 |
| Kule | 2021 | USA | 1/2 and 3 |
| Le | 2019 | Canada | 1/2 and 3 |
| Lee | 2015 | Korea | 1/2 |
| Lian | 2017 | Australia | 3 |
| Lim | 2017 | Korea | 1/2 and 3 |
| Limchareon | 2018 | Thailand | 3 |
| Limchareon | 2016 | Thailand | 1/2 |
| Linehan | 2020 | Canada | 1/2 |
| Lum | 2021 | USA | 1/2 and 3 |
| Mackay | 2018 | Canada | 3 |
| Maloney | 2020 | USA | 3 |
| Maloney | 2017 | USA | 3 |
| McCrary | 2017 | USA | 1/2 and 3 |
| McCrary | 2016 | USA | 3 |
| McVicar | 2015 | Canada | 3 |
| Menegozzo | 2019 | Brazil | 1/2 |
| Miller | 2017 | USA | 1/2 and 3 |
| Miller | 2016 | USA | 1/2 and 3 |
| Miner | 2015 | USA | 1/2 and 3 |
| Moak | 2014 | USA | 3 |
| Mouratev | 2013 | USA | 3 |
| Mullen | 2018 | USA | 3 |
| Nausheen | 2020 | USA | 3 |
| Nelson | 2017 | USA | 3 |
| Nitsche | 2021 | USA | 3 |
| Nourkami-Tutdibi | 2021 | Germany | 3 |
| Nuin | 2020 | Spain | 1/2 |
| Ochoa | 2019 | USA | 1/2 and 3 |
| Olszynski | 2020 | Canada | 1/2, 3 and 4 |
| Olszynski | 2018 | Canada | 1/2 and 3 |
| Oveland | 2013 | Norway | 1/2 and 3 |
| Parikh | 2018 | USA | 1/2 |
| Park | 2019 | Korea | 1/2 and 3 |
| Pazeli | 2018 | Brazil | 3 |
| Peyrony | 2018 | France | 3 |
| Poland | 2018 | USA | 1/2 and 3 |
| Potter | 2019 | USA | 1/2 |
| Reed | 2016 | USA | 1/2 and 3 |
| Risler | 2021 | USA | 1/2 and 3 |
| Russell | 2014 | USA | 3 |
| Schmidt | 2021 | Germany | 1/2 and 3 |
| Schneider | 2021 | Austria | 3 |
| Serrao | 2017 | Italy | 3 |
| Sevak | 2018 | USA | 1/2 and 3 |
| Shafqat | 2015 | UK | 1/2 and 3 |
| Shafqat | 2021 | UK | 3 |
| Shah | 2020 | USA | 1/2 |
| Shah | 2019 | Switzerland | 1/2 |
| Sheehan | 2010 | USA | 3 |
| Shmueli | 2013 | Israel | 1/2 and 3 |
| Shokoohi | 2016 | USA | 1/2 and 3 |
| Situ-LaCasse | 2019 | USA | 1/2 and 3 |
| Situ-LaCasse | 2021 | USA | 1/2 and 3 |
| Slomer | 2017 | Canada | 3 |
| Steinmetz | 2018 | Canada | 1/2 and 3 |
| Steinmetz | 2016 | Canada | 3 |
| Stellar | 2014 | USA | 3 |
| Stokke | 2014 | Norway | 3 |
| Sweetman | 2013 | Australia | 1/2 and 3 |
| Taksoe-Vester | 2018 | Denmark | 3 |
| Tolsgaard | 2015 | Denmark | 3 |
| Torabi | 2021 | USA | 1/2 |
| Tshibwabwa | 2016 | Barbuda | 1/2 and 3 |
| Udrea | 2017 | USA | 1/2 and 3 |
| Valenciaga | 2021 | USA | 1/2 |
| Vitto | 2016 | USA | 3 |
| Vyas | 2018 | USA | 4 |
| Walrod | 2019 | USA | 1 and 3 |
| Walrod | 2018 | USA | 3 |
| Weiskittel | 2021 | USA | 1/2 and 3 |
| Wong | 2011 | UK | 1/2 and 3 |
| Wong | 2021 | Hong Kong, China | 1/2 and 3 |
| Yan | 2018 | Hong Kong, China | 3 |
| Zawadka | 2019 | Poland | 1/2 |
| Zhao | 2021 | China | 1/2 |
